# A compact C-band FLASH electron linear accelerator prototype for the VHEE SAFEST project

**DOI:** 10.3389/fonc.2025.1516576

**Published:** 2025-03-06

**Authors:** Lucia Giuliano, David Alesini, Fabio Cardelli, Martina Carillo, Enrica Chiadroni, Massimiliano Coppola, Giacomo Cuttone, Alessandro Curcio, Angelica De Gregorio, Roberto Di Raddo, Luigi Faillace, Stefano Farina, Luca Ficcadenti, Daniele Francescone, Gaia Franciosini, Giovanni Franzini, Alessandro Gallo, Marco Magi, Giorgio S. Mauro, Andrea Mostacci, Luigi Palumbo, Vincenzo Patera, Francesca Perondi, Massimo Petrarca, Stefano Pioli, Romolo Remetti, Alessio Sarti, Angelo Schiavi, Bruno Spataro, Giuseppe Torrisi, Alessandro Vannozzi, Mauro Migliorati

**Affiliations:** ^1^ Scienze di Base e Applicate per l'Ingegneria (SBAI) Department, Sapienza University of Rome, Rome, Italy; ^2^ Sec. Roma1 of Istituto Nazionale di Fisica Nucleare, Rome, Italy; ^3^ Laboratori Nazionali di Frascati of Istituto Nazionale di Fisica Nucleare (LNF-INFN) Frascati, Frascati, Italy; ^4^ Laboratori Nazionali del Sud of Istituto Nazionale di Fisica Nucleare Catania, Catania, Italy; ^5^ Physics Department, Sapienza University of Rome, Rome, Italy

**Keywords:** FLASH linac, C-band, FLASH therapy, RF design, beam dynamic analysis

## Abstract

FLASH therapy, a novel cancer treatment technique, aims to control tumor growth, sparing the healthy tissue from radiation damage and thus increasing the therapeutic ratio. Translating FLASH therapy into clinical practice, especially for treating deep-seated tumors, necessitates achieving Very High-Energy Electron (VHEE) levels within the 50-250 MeV range. In 2022 Sapienza University, in collaboration with INFN, launched the SAFEST project, a compact C-band 100 MeV Ultra-High Dose Rate (UHRD) radiation source for the treatment of deep-seated tumors, which was partially funded by Italian PNRR (Next Generation EU). A C-band linac prototype at lower energy, with an electron pulse of 100 nC and repetition frequency <200 Hz, is being developed to test the key choices and technology of a VHEE machine. This paper provides insights into the design strategy of the prototype, discussing the optimization of the main RF and electron beam parameters. The expected dose profiles are also shown and discussed. The progress of this innovative linac represents a step forward in the realization of a C-band compact FLASH VHEE source for cancer treatment.

## 1 Introduction

Radiotherapy (RT) is an effective tool used in more than half of all cancer treatments. Over the past decades, personalized treatment modalities have been developed thanks to technological and biological advancements. However, success in fighting cancer is still constrained by complications in normal tissues and by radiation-induced side effects. These include acute effects, such as inflammation in early-responding tissues (e.g., skin), and late effects, like radiation-induced necrosis and functional loss in late-responding tissues (e.g., brain). Consequently, enhancing the therapeutic ratio, namely the relationship between the probability of tumor control and the likelihood of normal tissue damage, remains the primary goal of modern cancer research.

In the last decade, the investigation of radiobiology at Ultra High Dose Rate (UHDR) has brought a new avenue to the forefront: the so-called “FLASH effect” (1). *In-vivo* experiments under UHDR have shown a significant differential impact on tumors versus normal tissue: healthy tissue toxicity (side effects) is reduced, while tumor damage remains unchanged. If these results are confirmed, the FLASH effect could revolutionize RT. Even if the research in the FLASH regime is still in its early stages, studies involving electron beams [e.g., refs. (2–6)] have demonstrated promising results for clinical application. Early trials in humans (7) and domestic animals (8, 9) are underway, confirming the technique’s feasibility while also revealing challenges and the high interest within the medical community.

It remains unclear whether the mechanism behind the FLASH effect lies in differential DNA damage between tumor and healthy cells, in other cellular components (proteins, lipids, membranes, etc.), or if it involves a more complex interaction at the tissue or organ.

Clinical research facilities are too limited to explore this area thoroughly. The parameter space for FLASH therapy is vast, and a systematic investigation is necessary to identify the optimal conditions for future treatments.

A key topic of discussion in electron RT is whether VHEE could facilitate the clinical application of FLASH, as they have the potential to deliver UHDRs while penetrating deep-seated tissues. However, no existing prototypes currently meet hospital requirements because they are based on high-energy accelerators existing in large facilities. Our challenge is developing a compact system that is easy to operate in a hospital and can achieve electron pulses typical of the UHDR for FLASH, as reported in **Table 1** (10, 11).

**Table 1:** Main parameters for FLASH irradiation.

Symbol	Description	Value
PRF	Pulse repetition frequency	>100 Hz
$t_{p}$	Electron pulse width	0.1-4.0 μs
$t_{i}$	Total irradiation time	<100 ms
$\bar{\dot{D}}$	Time-averaged dose rate	>100 Gy/s
$\dot{D_{p}}$	Dose-rate in a single pulse	$>10^{6}$ Gy/s
$D_{p}$	Dose in a single pulse	>1 Gy

## 2 VHEE beams for radiotherapy

### 2.1 FLASH-VHEE therapy potential

The use of electrons in the 50–250 MeV energy range for the treatment of deep-seated tumors has been explored in previous studies demonstrating that VHEE treatments are competitive with conventional radiotherapy and particle therapy (12–15).

**Figure 1:**
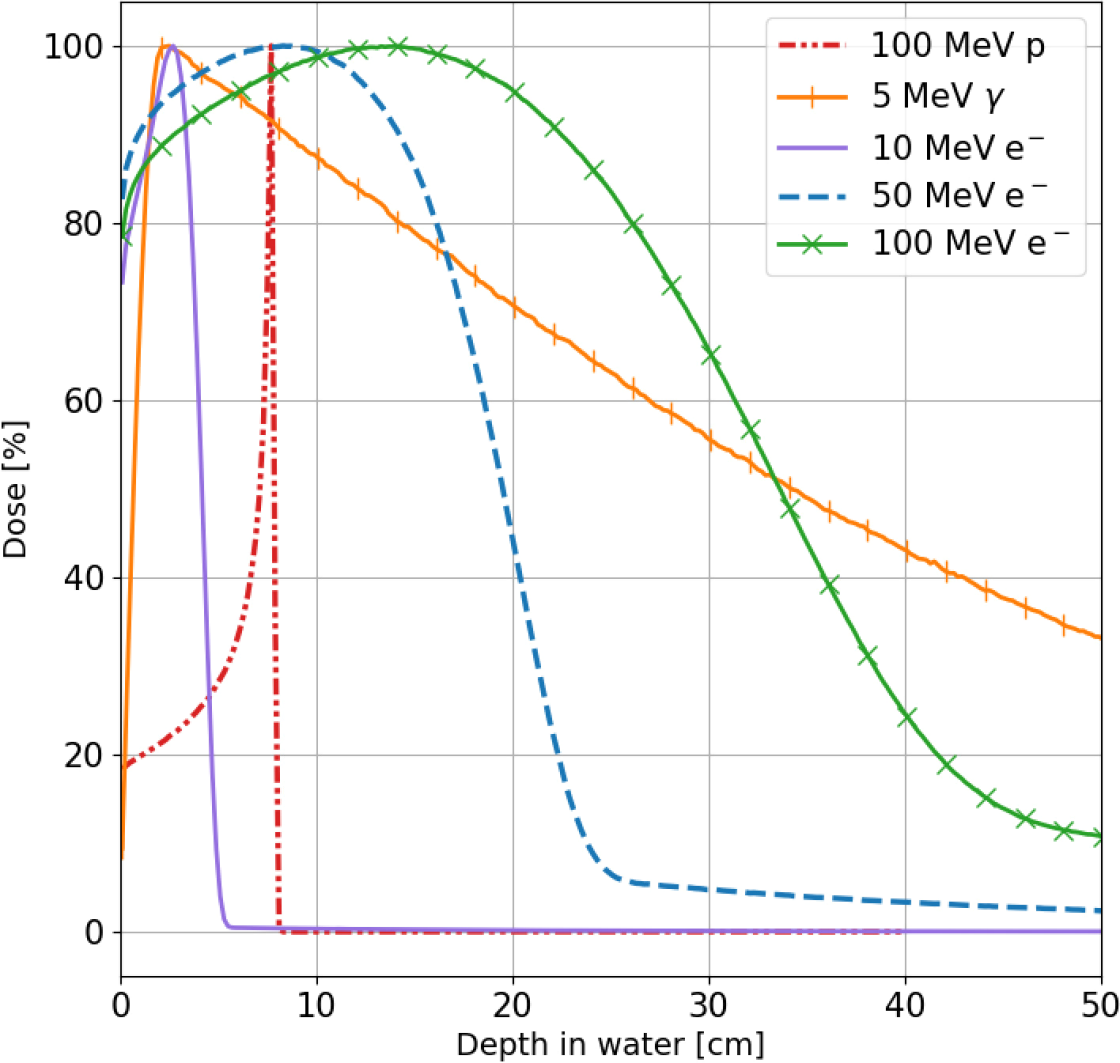
Percentage dose profiles along the beam axis for 10 MeV (violet line), 50 MeV (blue line) and 100 MeV (green line) electrons, 5 MeV photons (orange line) and 100 MeV protons (red line) in a water phantom obtained using FRED Monte Carlo software (16–18).

From a physical perspective, electron dose delivery represents a compromise between that of protons and photons. Due to their lower mass compared to protons, electrons exhibit a broader Bragg Peak (BP), as illustrated in **Figure 1**. However, as charged particles, electrons undergo multiple scattering interactions, unlike photons, leading to less conformal lateral dose deposition.

The broader percentage depth dose curve (PDD) of VHEE does not have the selectivity potential of other RT-charged beams as proton or carbon, but allows to overcome two main limitations of UHDR irradiation with hadrons and photons. There is no need to change the beam energy to cover a large target volume (electrons provide a naturally spread-out BP), and this helps in providing the required high dose rate. There is no need to implement strict safety margins on the patient positioning as the longitudinal absorbed dose distribution does not exhibit any sharp fall.

It is worth mentioning that recent research has shown the additional possibility of tailoring and/or narrowing the PDD of VHEE using suitable magnetic systems to tightly focus the electron beams (19). In fact, an appealing feature of VHEE is the magnetic rigidity smaller than that of protons, which allows easier and faster magnetic bending of the beam when delivered in an active scanning, as in the pencil beams approach.

## 3 SAFEST Project: a 100 MeV compact C-band linac

### 3.1 Linac frequency choice

Electron medical accelerators are based on accelerating structures with a resonant frequency in the (S-C-X) band, operating in either Traveling Waves (TW) or Standing Waves (SW) modes. Different technological solutions have been adopted (20) depending on the operating resonant frequency, which represents one of the most RF-relevant parameters to choose when designing a linac. In particular, the scaling laws (21, 22) indicate that, as the frequency of RF cavities increases, it becomes possible to achieve a certain electron beam energy with a shorter accelerator length for a given power. This can be attributed to two main factors: higher shunt impedance per unit length and a higher maximum attainable electric field strength. Moreover, it’s known that the maximum accelerating field in RF structures is limited by the RF breakdown effect, which can result in significant damage to the structure. According to the empirical laws (23–25), a higher frequency of operation allows a higher breakdown limit and a higher accelerating gradient. As a consequence, there are advantages in utilizing higher frequencies. The primary benefit lies in the compactness of the accelerator, resulting in reduced size and weight.

In the past, significant progress was made in developing warm X-band traveling wave (TW) structures, achieving an accelerating gradient of 100 MV/m (26–28). These X-band developments were mainly driven by the need for highly efficient future accelerators for high-energy physics. More recently, C-band warm TW structures achieved a gradient of 50 MV/m (29–33), mainly used in FEL and Compton Sources. Higher gradients, of the order of 100 MV/m, were achieved with C-band structures operating at cryo temperatures (77 K) (34, 35). Also, in industrial and medical applications, there are advantages in utilizing higher frequencies. In this case, SW structures are preferred to accelerate low-energy electrons (20, 36) despite their lower energy efficiency.

For a specific application, the choice of the frequency is crucial to consider certain trade-off factors when going to higher frequencies. Indeed, from the definition of the shunt impedance per unit length:


$$R_{sh}=\frac{V^{2}}{P_{cav}}\frac{1}{L_{str}}$$

with *V* the accelerating voltage, $P_{cav}$ the power dissipated in the cavity walls, and $L_{str}$ the linac length, it’s apparent that with higher frequency, there is a higher power efficiency.

Nevertheless, other parameters are worsened, such as a lower Q-value and a reduced power dissipation capability of the accelerator structure. Of course, some of these challenges can be overcome through a meticulous design of the cooling system, for example, and high-precision machining and polishing. Finally, in the case of high peak current, the radius of the iris of the accelerating cells plays an important role in the total charge transmission efficiency of the beam (21): a small iris radius typical of high-frequency structures results in a higher number of charges hitting the iris metallic surface, particularly in the structure at low energy where particles are captured.

Taking into account the above considerations, and based on our experience with C-Band technology (31–33), we proposed for the SAFEST Project a compact C-band linac, operating at the frequency of 5.712 GHz, as the best compromise which combines a high shunt impedance $R_{sh}$ with an optimized transmission efficiency allowing a high peak current and UHDR electrons pulses.

### 3.2 SAFEST linac

In the SAFEST linac (11, 37) electrons are generated by a pulsed DC thermionic gun and injected into a 70 cm long SW structure, bi-periodic, working in *π*/2 mode. The beam is accelerated up to the energy of 10 MeV, and with two 1 m long TW structures, it reaches the energy of 100 MeV. A 45 MW klystron powers the accelerating system and foresees the use of a pulse compressor. Beam dynamics studies show that it’s possible to accelerate an electron pulse with a charge of 100 nC at the energy of 100 MeV. The accelerating linac is shorter than 3 m, and the system, including vacuum pumps and diagnostic devices, does not exceed 4 m.

The choice of a SW structure as the first accelerating section of the linac is due to several reasons. Indeed, the SW field configuration offers the advantage of maintaining a stable and well-focused particle beam without requiring additional focusing magnets, such as solenoids. This is due to the extra focusing effect of the non-synchronous accelerating field components (backward waves) of a SW structure (38, 39), especially in the low-beta bunching section where electrons are generated with low kinetic energy. Further, it’s possible to feed the structure with a single coupler located far from the cathode cell and the bunching sections, thereby avoiding any distortion of the accelerating field caused by the coupling window. Finally, the fixed phase advance per cell (here *π*/2), combined with appropriately designed cell length, optimizes the beam longitudinal capture.

## 4 C-band prototype @ Sapienza

### 4.1 Start of the project

The SAFEST project was partially funded in 2023 in the framework of the Italian PNRR plan (EU Next Generation) with a budget limited to the construction of a prototype in which the key components of the system, in particular the hybrid scheme SW and TW, can be tested at lower energy. The construction of the prototype in the Sapienza Campus is expected to be completed at the beginning of 2026. The area where the prototype will be located has, at the moment, a radio-protection constraint limiting the electron energy to 24 MeV. Due to the above limitations, the prototype cannot be used at its potentially extreme performance. Accordingly, we are planning to construct a 24 MeV prototype able to deliver UHDR pulses typical of the FLASH regime, which will allow us to test the combination of SW and TW sections in a compact system. The facility will provide a flexible platform for the development and test of innovative devices for precise measurement, monitoring, and manipulation of electron beam parameters under FLASH conditions and for conducting radiobiology experiments with both *in-vitro* and *in-vivo* samples.

### 4.2 Prototype description and design

#### 4.2.1 Beam parameters and schematic layout

**Figure 2:**
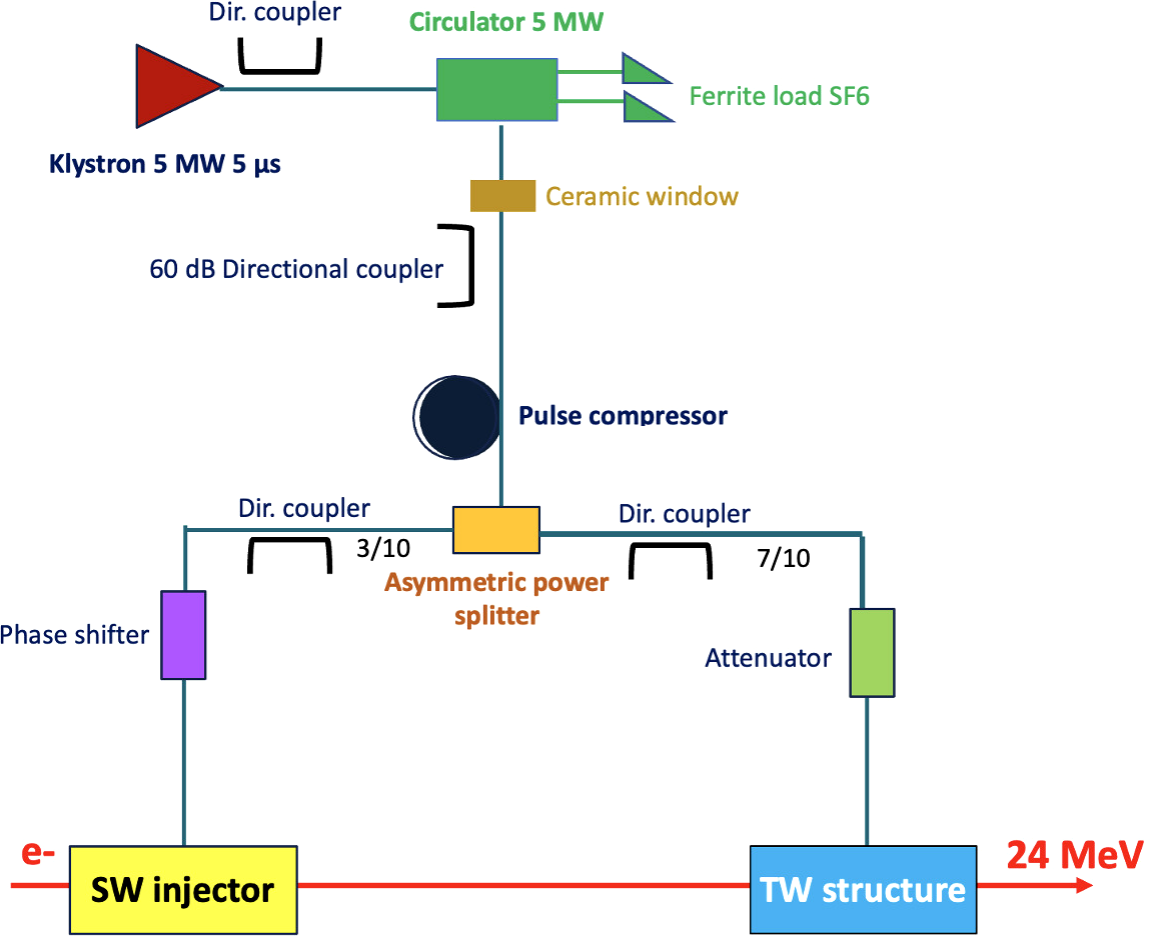
Layout of VHEE FLASH linac prototype.

The layout of the proposed prototype is shown in **Figure 2**, highlighting the RF power distribution. The accelerating structures are powered by a 5 MW klystron with an RF pulse length of 5 µs. The klystron output feeds a pulse compressor (40) to obtain a shorter pulse length with a peak power of about 25 MW. This pulse enters a power splitter which distributes the available power asymmetrically: the standing wave structure receives 30% of the maximum available power. At the same time, the 70% is directed to the traveling wave structure. The circulator is needed to avoid damage to the klystron due to uncontrolled backward power from the SW cavity. The phase shifter guarantees the proper phase relation between the two structures, while the attenuator allows reducing the accelerating gradient in the TW structure to stay at the nominal energy of 24 MeV. Additionally, the klystron output power can be varied by an input control signal: a small negligible fraction of the klystron output taken from the directional coupler shown in the upper part of the figure (before the circulator) is compared with a reference signal and used to control the klystron input signal.

As shown in **Table 2**, where the main parameters of the linac prototype are reported, the first SW linac captures and accelerates a 1 µs pulse current of 100 mA, up to the energy of about 11 MeV. The beam reaches then an energy of 24 MeV in the following traveling wave section. The total length of the linac is about one meter and a half.

**Table 2:** Linac prototype main parameters.

Linac parameters	
Frequency	5.712 GHz
Klystron Power	5 MW
Repetition frequency	< 200 Hz
RF pulse width	5 *µ*s
Peak power after compression	24.4 MW
Total linac length	150 cm
Nominal beam energy (loaded)	24 MeV
Current Pulse *I_b_*	100 mA
Pulse current duration	1 *µ*s
Pulse charge	100 nC
Pulse compressor	
Operating mode	*TE*_114_
Unloaded Quality Factor *Q*_0_	134000
Coupling coefficient *β_sled_*	3
RF input pulse length	5 *µ*s
SW structure	
Structure length *L_SW_*	69 cm
Shunt Impedance *R_SW_*	116 MΩ/m
Quality factor *Q_SW_*	10178
Mode of operation	Bi-periodic *π*/2
N of accelerating cells *N_SW_*	27
Coupling cells length	3 mm
Iris radius	3 mm
Filling time	0.220 *µ*s
Coupling coefficient *β_SW_*	1.58
TW structure	
Structure length *L_TW_*	43 cm
Number of cells	24
Shunt Impedance *R_TW_*	107 MΩ/m
Quality factor *Q_TW_* (cell)	10630
Type	Constant Impedance
Operation mode	$\frac{2}{3}\pi$
Iris radius	5 mm
Filling Time	0.143 *µ*s
Group velocity *v_g_*	0.01c*

(*) *c* = speed of light.

#### 4.2.2 Pulse compressor

A spherical cavity RF pulse compressor – selected because of its compactness and relative ease of fabrication – is adopted to compress the 5 MW, 5 *µ*s RF pulse coming from the klystron.

**Figure 3:**
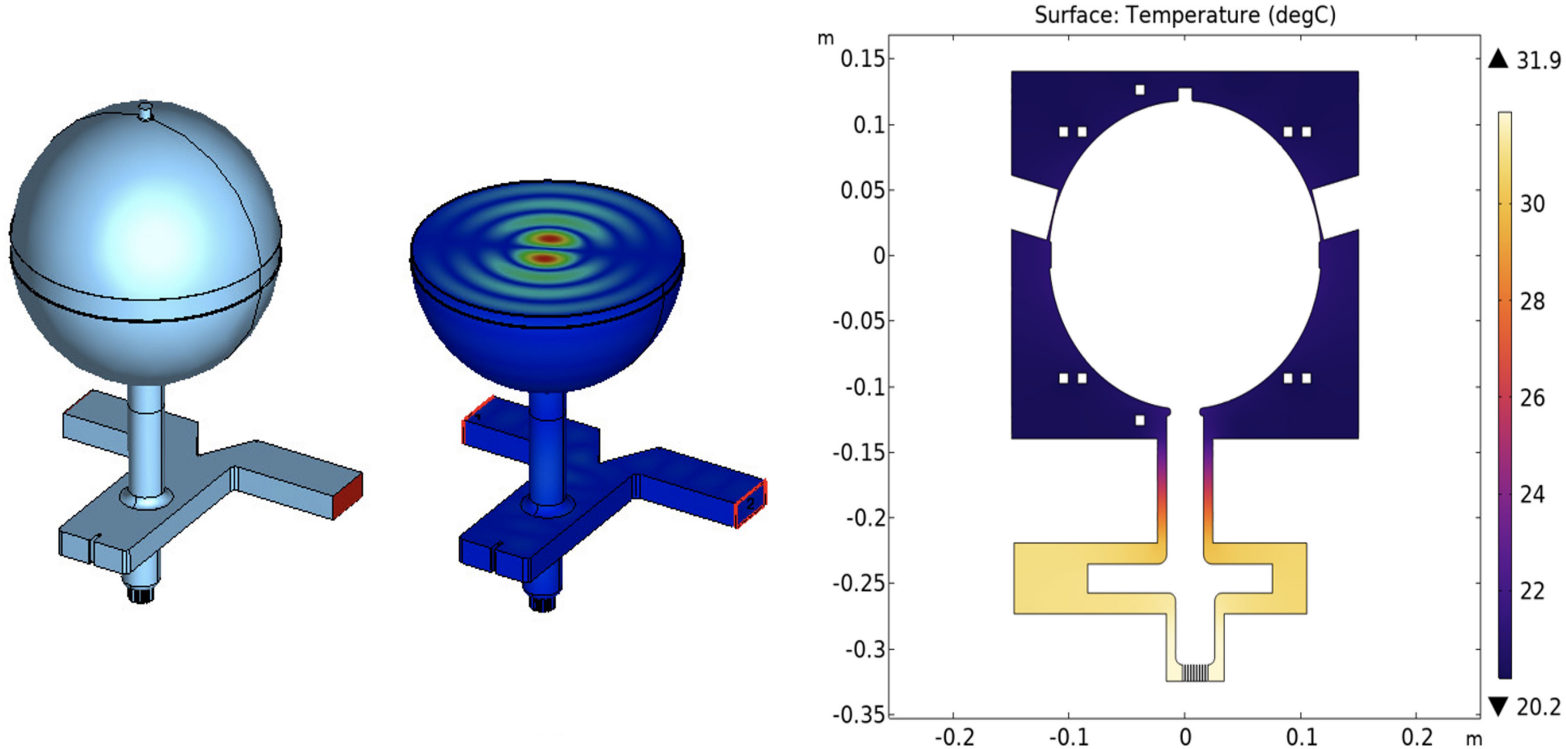
Spherical cavity pulse compressor: RF model (left), 2D cut showing the TE_114_ electric field (center), temperature distribution along the cut of the pulse compressor structure (right).

The spherical cavity pulse compressor, visible in **Figure 3**, is composed of a 3 dB coupler (also acting as a circular polarizer, converting the input TE_10_ mode into two, 90-deg shifted, circular TE_11_ output modes) and a single spherical energy storage cavity.

These two subsystems were first designed separately and then assembled to obtain the complete device. For the spherical cavity, two degenerated TE_114_ modes (see **Figure 3**, center) have been chosen for operation because of their high unloaded quality factor, *Q*_0_ = 134 × 10^3^.

The coupling coefficient $\beta_{sled}$ of the pulse compressor is calculated to be about 3. The operating frequency can be tuned in two ways: before brazing, by machining a circular ridge placed in the sphere equator (removal of 1 mm in ridge thickness corresponds to a frequency shift of about −2.5 MHz); after brazing, by employing eight push-pull tuners (a penetration of 0.5 mm for the eight tuners corresponds to a frequency shift of about +0.4 MHz). A summary of some main parameters of the pulse compressor is reported in **Table 2**.

Preliminary COMSOL thermal simulations (41) have been performed on a simplified mechanical model. Considering an input water temperature equal to 20°C and water flux of 20 $\frac{L}{min}$ , the temperature distribution visible in the right-hand side of **Figure 3** has been obtained. It can be seen that the optimized cooling system allows a temperature distribution close to the input water temperature in the spherical cavity area: further COMSOL structural simulations show that this temperature distribution avoids undesired structure deformations, thus having a negligible effect on resonant frequency value.

#### 4.2.3 Standing wave structure

**Figure 4:**
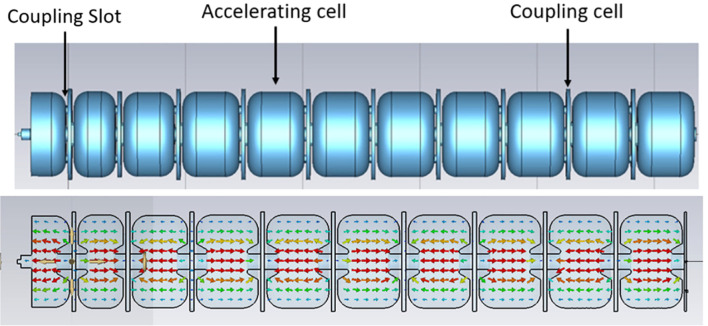
RF model of the SW structure with *π*/2 accelerating field operation mode. The first 3 low *β* sections, and 8 (out of 24 of the final design) *β* = 1 sections are shown.

The C-band SW bi-periodic structure operates in a *π/*2-mode. It alternates coupling cavities, with no electric field, and accelerating cavities in which the electric field is maximum, as shown in **Figure 4**. Off-axis magnetic coupling slots are used to connect the accelerating cells with the coupling ones so that the electromagnetic energy can flow through the structure during the pulse generated by the power source.

A detailed analytical and numerical study with CST Microwave Studio Suite (42) has been carried out. The unit cell, which represents the basic device that was optimized, consists of an accelerating cell, two half-coupling cells, and two pairs of slots. The condition of a perfectly conducting surface (PE) at the structure boundaries was imposed to evaluate the resonant frequency and the main RF parameters. The shunt impedance was evaluated considering the copper wall conductivity.

As a first step in the linac design, after fixing the accelerating cell length *L* according to:


(1) $$L=\frac{\beta \text{λ}}{2},$$

with *β* the relativistic factor and λ the electric field wavelength, we determined the cell’s diameters in such a way as to have the right resonance frequency of 5.712 GHz in each cell.

According to Equation 1, the first part of the linac presents a bunching section, composed of three cavities with different lengths, which takes into account the initial non-relativistic speed of the electron beam (*β* << 1). In these cells, a higher peak electric field improves the beam capture (43). After the first three cells, the beam becomes relativistic, and the cells maintain the same length because the velocity variation of the particles is negligible. The first cavity of the bunching section is a half-accelerating cell with an end plate where the electron gun is inserted.

The unit accelerating cell presents a nose-cone structure, visible in the lower part of **Figure 4**, to maximize the efficiency of the acceleration. Indeed, the nose cone allows the localization of a very high electric field on the axis, creating an efficient beam acceleration. Several iterations have been performed to choose the proper geometry of the two nose cones to achieve high shunt impedance and a high electric field in the center of the cell.

Another important step in the design of the accelerating structure is the optimization of the quality factor $\left(Q_{SW}\right)$ , which represents the ratio of stored power to the power dissipated at the cavity walls. To this aim, the curvature on the top of the cavity has been varied until reaching an acceptably high value. In general, a higher quality factor and a higher shunt impedance mean higher machine performances.

Finally, the iris radius has been obtained as a compromise between a high shunt impedance per unit length $R_{SW}$ and reasonable particle transmission. A low value of this radius increases $R_{SW}$ but also leads to losses caused by the particles hitting the cavity walls that could activate the copper, creating radioprotection concerns. The chosen values of $Q_{SW}$ , $R_{SW}$ , and $R_{b}$ are shown in **Table 2**.

The power feeds the cavities using a tapered RF waveguide through a hole in the wall of the central accelerating cell. This location is chosen because the central coupling, due to the symmetry, allows reducing the excitation of half of the linac resonant modes. The dimensions of the coupling hole between the waveguide and the linac have to be suitably chosen to obtain a proper balance between the input and the reflected power, taking into account also the phenomenon of the beam loading (21). Indeed, the waveguide-to-linac coupling coefficient $\beta_{SW}$ must be optimized to minimize the reflected RF power when the electron beam is accelerated. The formula we used as a reference is the following:


(2) $$\beta_{SW}=1+\frac{P_{beam}}{P_{cav}}=1+\frac{I_{b}R_{SW}}{V_{cav}}$$

where $P_{beam}$ is the power delivered to the beam, and $V_{cav}$ is the net cavity voltage (on the crest of the accelerating wave) (21). The value obtained with Equation 2 is shown in **Table 2**.

**Figure 5:**
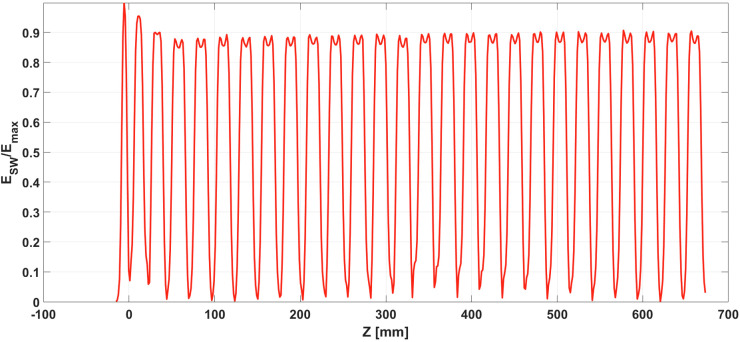
On-axis longitudinal electric field of the SW structure normalized to its maximum value.

**Figure 5** shows the amplitude of the longitudinal electric field obtained by CST along the axis and normalized to its maximum value. Of course, the final accelerating gradient depends on the available power. In the first 3 bunching sections, a higher field, which helps the capture process, can be noted (36). Also around the nose cones, the field is higher than in the center of the cells, while it vanishes in the coupling cells.

As already said, the SW field configuration offers the advantage of maintaining a stable and well-focused particle beam without requiring additional focusing magnets, such as solenoids (38, 39), as confirmed by beam dynamics simulations described in the next paragraph.

The simulated reflection coefficient *S*_11_ of the whole structure, which includes the tapered feeding waveguide, shows that the resonant frequency is 5712.7 MHz, resulting within the tunability range of the klystron (5708-5716 MHz). Additionally, we obtained that there are no longitudinal modes that can be excited inside this frequency range (± 4 MHz).

The main RF parameters of the SW structure are summarized in **Table 2**.

A small copper prototype, composed of five *β* = 1 cells was constructed in collaboration with SIT Sordina IORT Technology Spa and characterized at the Accelerator Laboratory of Sapienza University of Rome (44). In particular, the on-axis accelerating electric field was measured with the bead-pull technique. The tuning procedure provided a nearly uniform electric field distribution across the accelerating cells and, as expected, no field was detected in the coupling cells.

#### 4.2.4 Traveling wave structure

**Figure 6:**
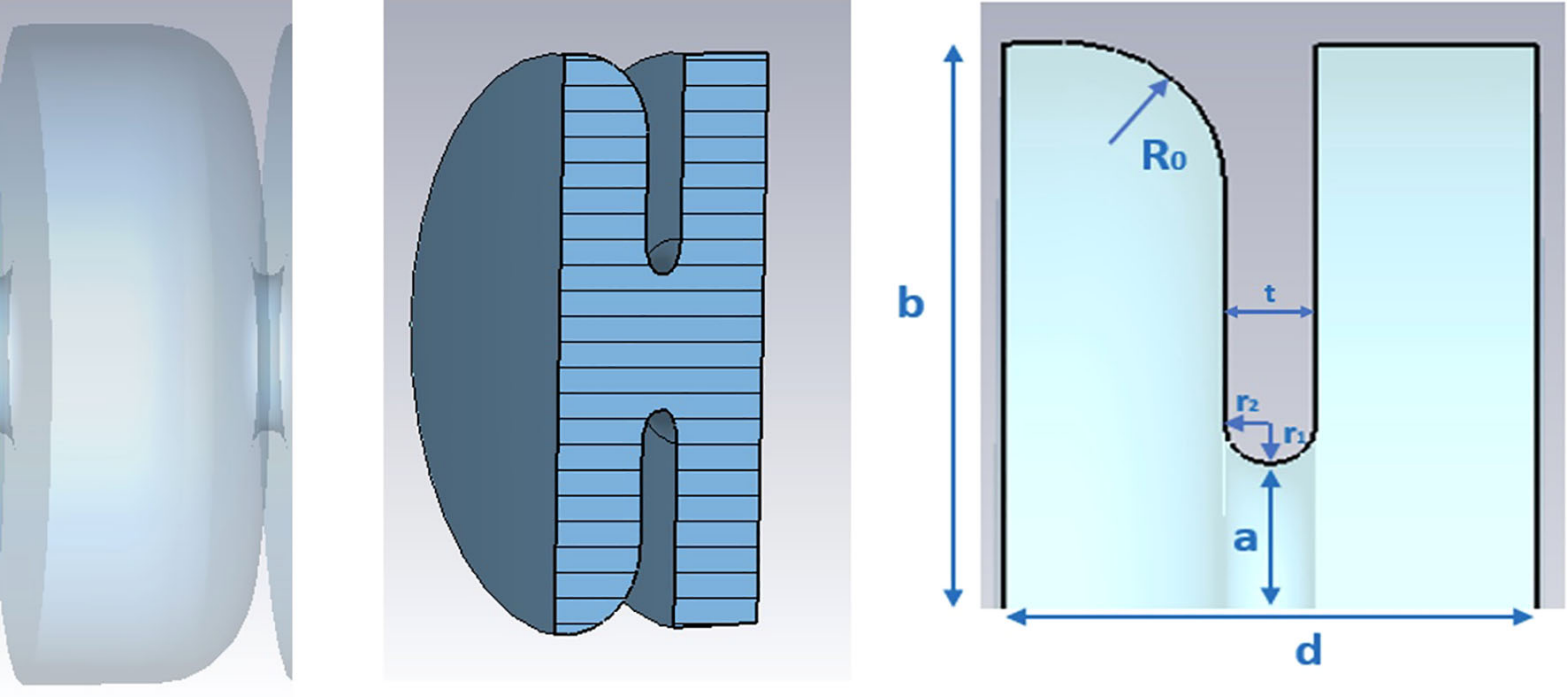
Single cell of the TW structure (left), perspective view of the CST simulated single structure (center), CST simulated geometry (right).

The traveling wave (TW) device is a C-band accelerating structure operating in a TM01-like mode with a $\frac{2}{3}\pi$ phase advance per cell, optimizing the acceleration process’s efficiency. Electromagnetic simulations and the design of the structure’s cells were carried out using CST Studio Suite, starting with the analysis of a single structure shown on the right-hand side of **Figure 6** and consisting of two half cells with proper boundary conditions. The left-hand side of the same figure shows the entire single cell of the TW system.

To simplify the in-house mechanical machining, we opted for the design of a cell geometry with left-right asymmetry. The rounding surface radius *R*_0_ was optimized to maximize the Q-factor, while the ratio between *r*_1_ and *r*_2_ was chosen to maximize the shunt impedance avoiding high-peak electric fields to prevent breakdown phenomena. Further, the size of the iris radius was chosen to achieve a high shunt impedance while maintaining a high group velocity. This ensures that the structure can be filled within a time frame compatible with the duration of the RF pulse.

**Figure 7:**
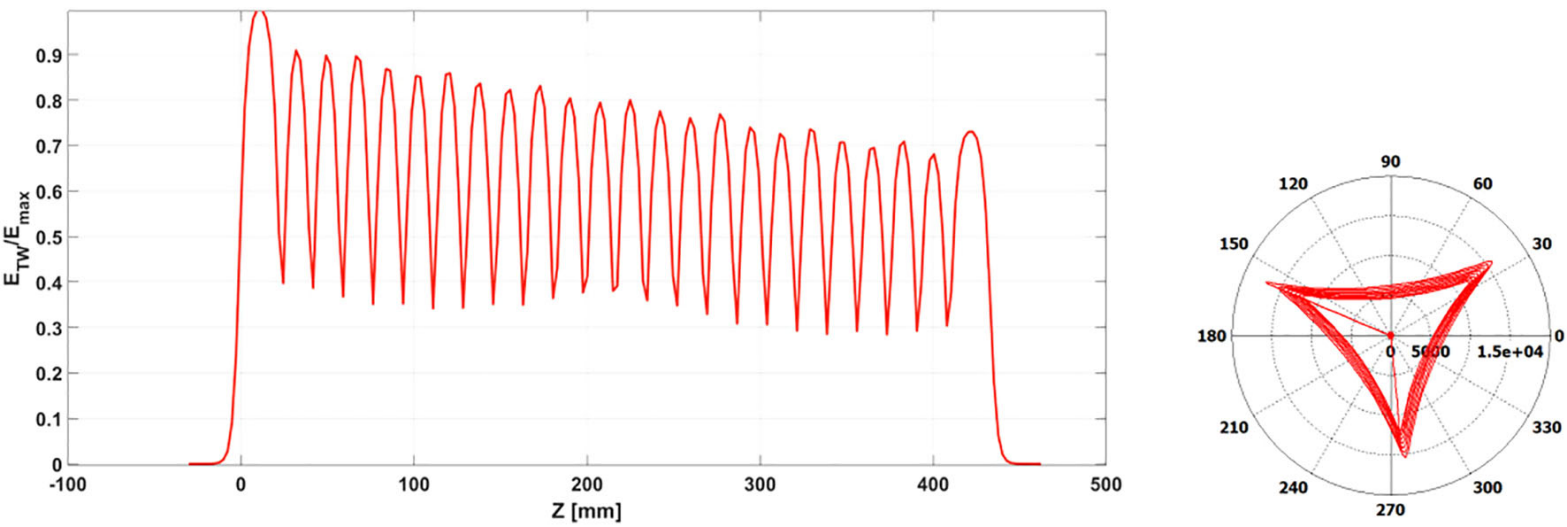
TW electric field normalized to its maximum value (left) and phase advance between cells after short circuit method optimization (right).

For the design of the input and output couplers we resorted to the short-circuit method (45), which allowed us to fine-tune the system to minimize the reflection coefficient at the waveguide input port for both the couplers. This approach ensures efficient power transfer into the structure while achieving a high degree of electric field flatness. The constant impedance of the structure inherently leads to some acceptable field attenuation as shown in **Figure 7**. One key finding during the optimization phase was that, due to the intrinsic asymmetry of the cells, the input and output couplers had to be designed with slightly different dimensions. As a result, the two couplers are not perfectly identical.

**Figure 8:**
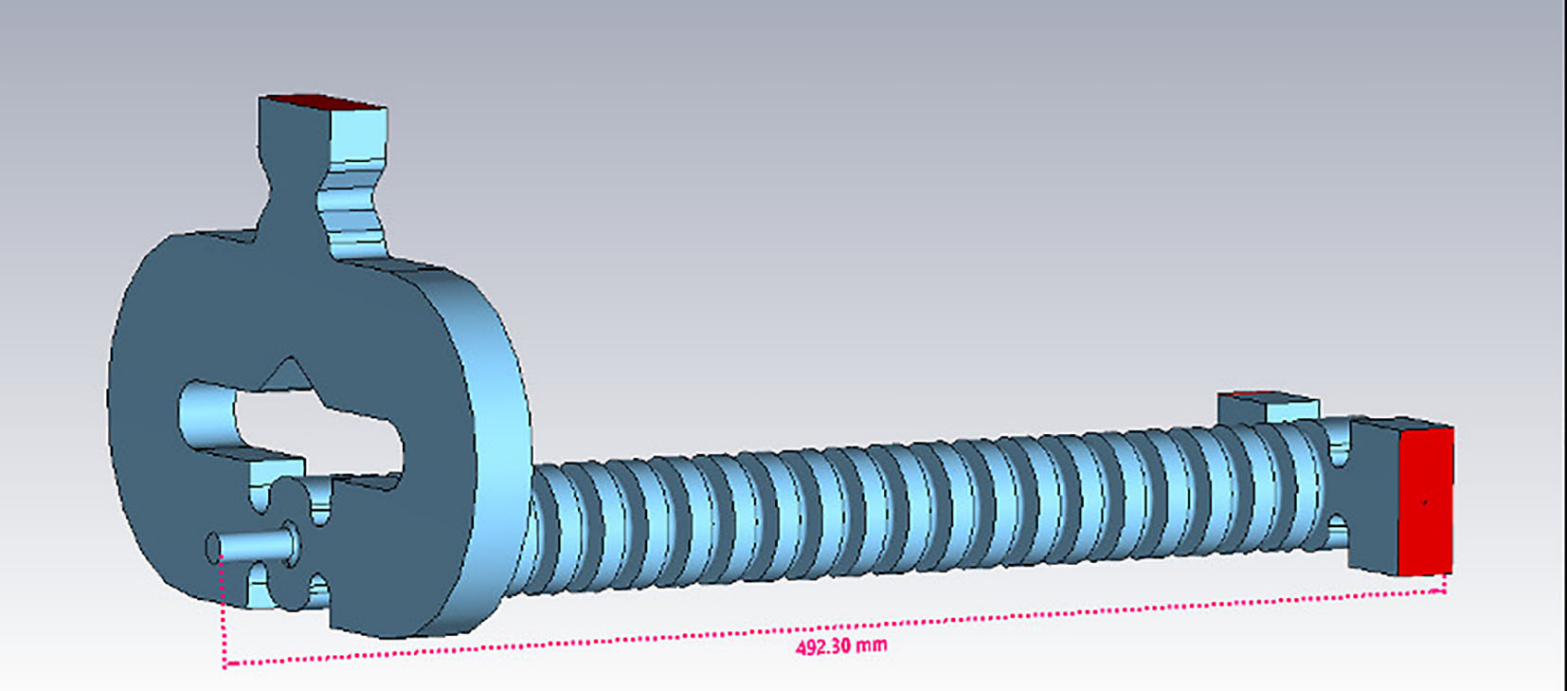
3D model of the TW structure. The feeding from the RF waveguide is obtained with a power splitter.

Power is fed into the accelerating structure through a splitter as shown in **Figure 8**, which is integrated into the input coupler. The splitter itself was carefully optimized to ensure maximum power transmission while preventing any mode crosstalk.

The main RF parameters of the TW structure are reported in **Table 2**

For this TW structure, two prototypes were designed, mechanically engineered and built in collaboration with Roma1 Section of INFN: the first one containing 13 accelerating cells with a maximum length of approximately 30 cm, and the second one with a length of about 50 cm containing 22 cells. The prototypes were useful to verify the mechanical precision of the in-house fabrication and the success of the brazing process performed at the Frascati National Laboratories of INFN. Low-power measurements, field mapping, and tuning process were performed on the prototypes to correct any mechanical errors and ensure the quality of the final structure.

### 4.3 Beam dynamics studies

The ASTRA (A Space Charge Tracking Algorithm) (46) code has been used to perform single bunch beam dynamics simulations for the C-band linac prototype.

In the following, we show the study of the transport efficiency of the beam current, the particle acceleration and the evolution of relevant beam parameters along the linac.

Furthermore, we present a semi-analytical study of the beam loading in the accelerating structures to determine the correct strategy to compensate for the energy spread along the train of accelerated electron bunches induced by the shape of the compressed RF pulse and by the self-induced electromagnetic fields.

#### 4.3.1 Electron pulse time structure

**Figure 9:**
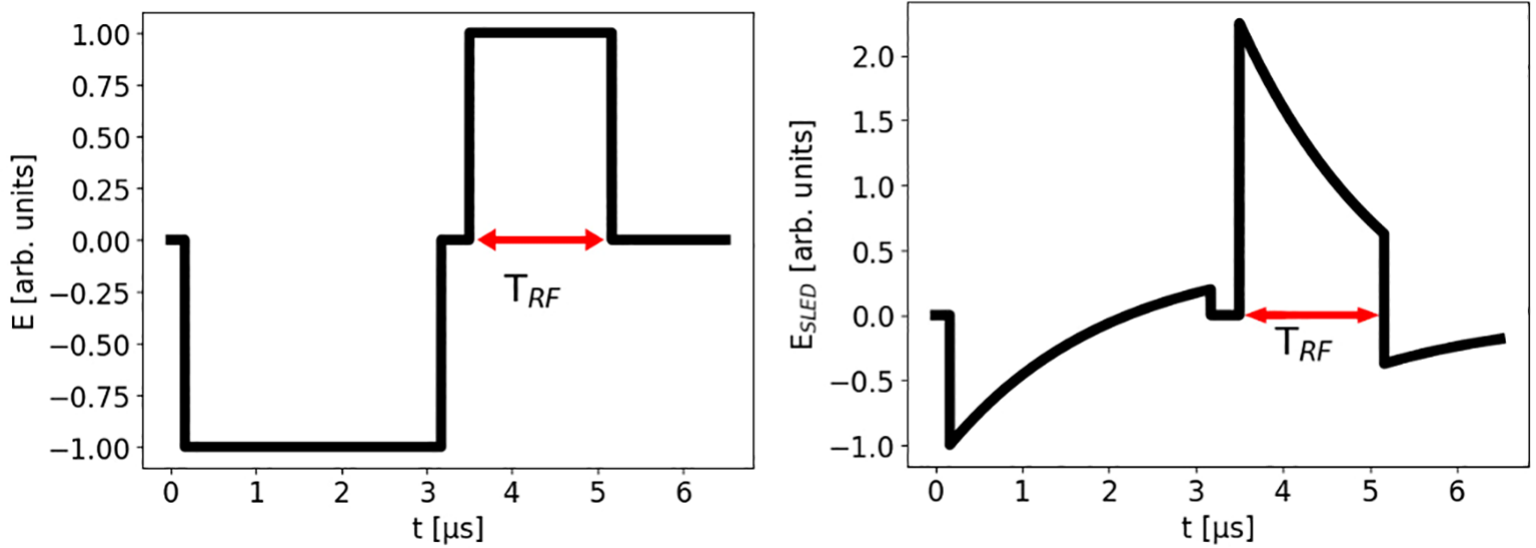
RF electric field before (left) and after compression (right).

As already stated, the squared 5 *µ*s pulses exiting the klystron at a repetition rate < 200 Hz are compressed inside the SLED cavity, as depicted in **Figure 9**. A critical aspect is the synchronization between the RF pulses in the two accelerating structures and the beam current pulse obtained by the DC emission from the thermionic cathode, which is pulsed to 1 *µ*s to obtain a current of 100 mA in 5712 bunches (each carrying about 18 pC). Optimal beam injection times can be found for the two structures, $t_{0,SW}$ and $t_{0,TW}$ respectively. Such a synchronization allows reducing the energy spread along the beam current pulse. In fact, the energy distribution along the current pulse is affected by the combination of beam loading and the shape of the compressed RF pulses, which modulates the accelerating field experienced by the electron bunches in the pulse. The shape of the compressed pulse consists in a prepulse, which is not used for acceleration, and a main pulse that starts in correspondence of a phase inversion imposed on the RF field at low power (see **Figure 9**). The time of phase inversion occurs $T_{RF}$ seconds before the end of the uncompressed pulse: indeed $T_{RF}$ denotes the length of the baseline of the main compressed pulse. The $\beta_{sled}$ coefficient determines the filling time of the SLED cavity and has a crucial role for the shape of the compressed pulse, i.e. for the induced energy spread. A lower $\beta_{sled}$ allows for slower decay of the compressed pulse on the scale of the beam current pulse length (1 *µ*s), at the expense of the maximum attainable peak power after compression.

#### 4.3.2 Beam capture and energy gain

**Figure 10:**
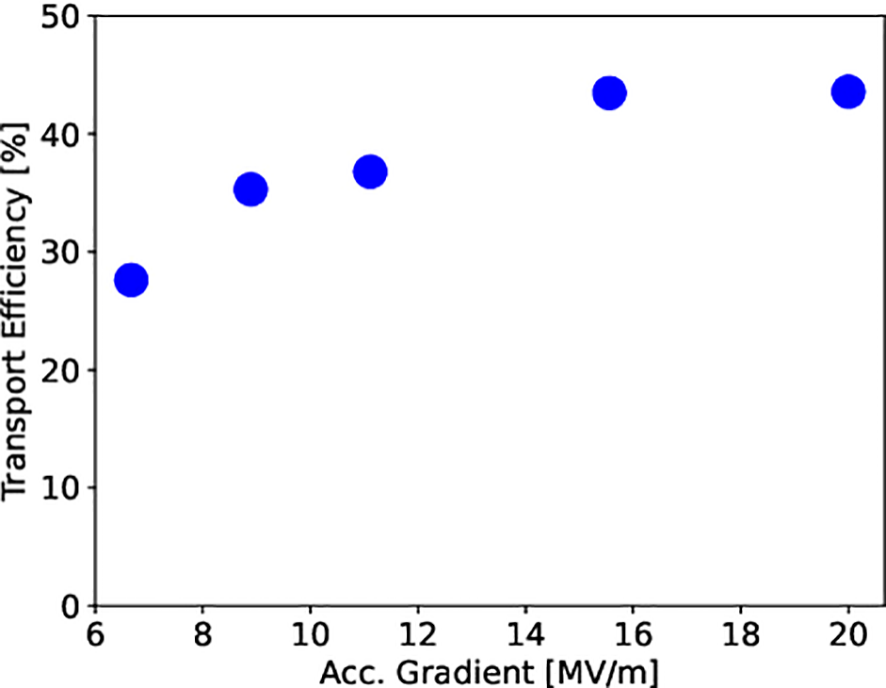
Efficiency of beam current transport from the cathode to the end of the SW structure for different average accelerating electric fields (i. e. accelerating gradients).

The first object of the simulation study is the transport efficiency from the cathode to the end of the accelerator, which is related to the beam capture at the entrance of the SW accelerating cavity. this transport efficiency has been determined versus the average accelerating electric field (also called accelerating gradient) reached in the SW structure. **Figure 10** shows the results of the ASTRA simulations.

A quasi-saturation level of about 44% is reached for an accelerating gradient greater than 15 MV/m. This means that in order to provide 100 mA of beam current for experiments at the end of the accelerator, about 230 mA must be emitted by the cathode.

**Figure 11:**
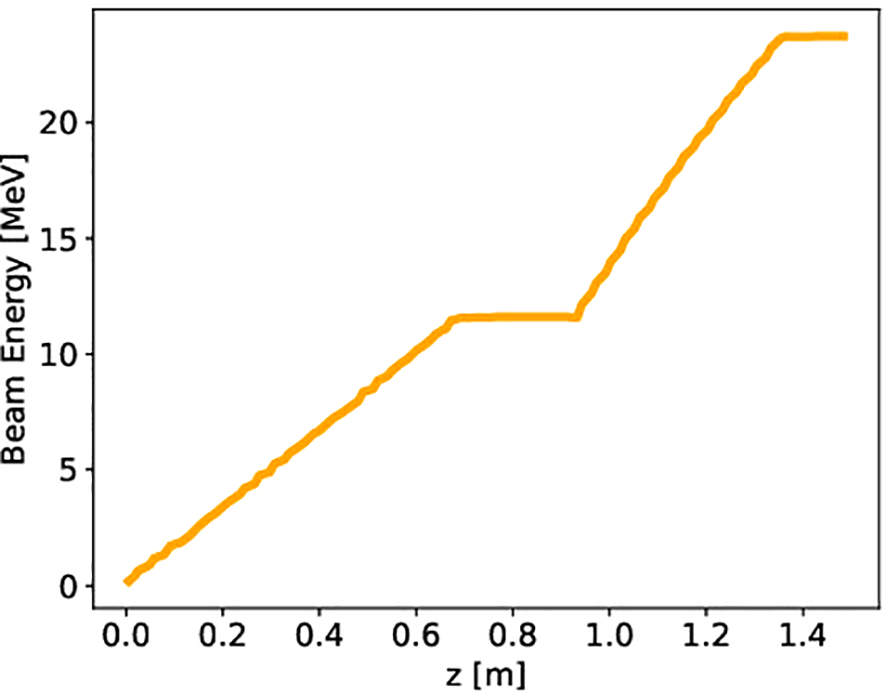
Energy gain for a single bunch traveling through the lattice made of an SW and a TW structure. Beam loading is considered for a current of 100 mA, reducing the net gain to ∼ 11 MeV in the SW cavity and to ∼ 13 MeV in the TW structure.

The desired final energy of the electron beam at the exit of the linac is 24 MeV. According to the simulations’ results, we can reach this value by accelerating the beam from a few tens of keV (emitted by the cathode) to ∼ 11 MeV in the SW structure, and then gaining an extra 13 MeV in the TW accelerating cavity, as shown in **Figure 11**.

Analytically, the mean energy gain $\text{Δ}W_{SW}$ in the SW cavity can be calculated using the following equation (36):


(3) $$\text{Δ}W_{SW}=\frac{2e\sqrt{R_{SW}L_{SW}\beta_{SW}P_{SW}}}{1+\beta_{SW}}-\frac{eR_{SW}L_{SW}}{1+\beta_{SW}}I_{b}$$

where *e* is the elementary charge. The first term in the right-hand side of the equation takes into account the external RF power feeding the structure, while the second term is due to the beam loading. If we consider the relevant parameters of the SW cavity reported in **Table 2**, in order to obtain an energy gain of about $\text{Δ}W_{SW}\;≃11$ MeV, and with a pulse current $I_{b}$ of 100 mA, we need an input cavity peak RF power $P_{SW}$ of 2.8 MW.

Moreover, the mean on-crest energy gain $\text{Δ}W_{TW}$ in the TW structure of ∼ 13 MeV can be calculated through the following equation and by considering an input peak RF Power $P_{TW}$ of 15.5 MW:


(4) $$\begin{array}{c} \text{Δ}W_{TW}=e\sqrt{2R_{TW}L_{TW}P_{TW}}\left(1-e^{-\frac{\pi \nu_{RF}L_{TW}}{v_{g}Q_{TW}}}\right)\sqrt{\frac{v_{g}Q_{TW}}{\pi \nu_{RF}L_{TW}}}\\ -\;\left(1-e^{-\frac{\pi \nu_{RF}L_{TW}}{v_{g}Q_{TW}}}\right)\frac{v_{g}Q_{TW}eI_{b}R_{TW}}{\pi \nu_{RF}} \end{array}$$

The relevant parameters of the TW structure are reported in **Table 2**.

The peak electric field value in the SW cavity is 40 MV/m. This choice was made to reduce the energy gain from 14 MeV to 11 MeV, in order to introduce the effect of beam loading in the ASTRA simulations. Therefore, this value should be understood as a loaded value, since the unloaded value of the peak electric field in the SW cavity is ≳ 50 MV/m. In other words, a peak electric field of ≳ 50 MV/m in the SW cavity can be achieved with the available RF power. However, due to the beam loading induced by the 100 mA beam current, this value will be reduced to 40 MV/m. Furthermore, the average accelerating field in the TW structure is chosen to be 26 MV/m, although the C-band structure can be powered in such a way as to obtain fields up to 40 MV/m. The reason for this choice was due to radiation protection limitations since the prototype linac can be operated with a maximum energy of 24 MeV.

#### 4.3.3 Transverse and longitudinal dynamics

**Figure 12:**
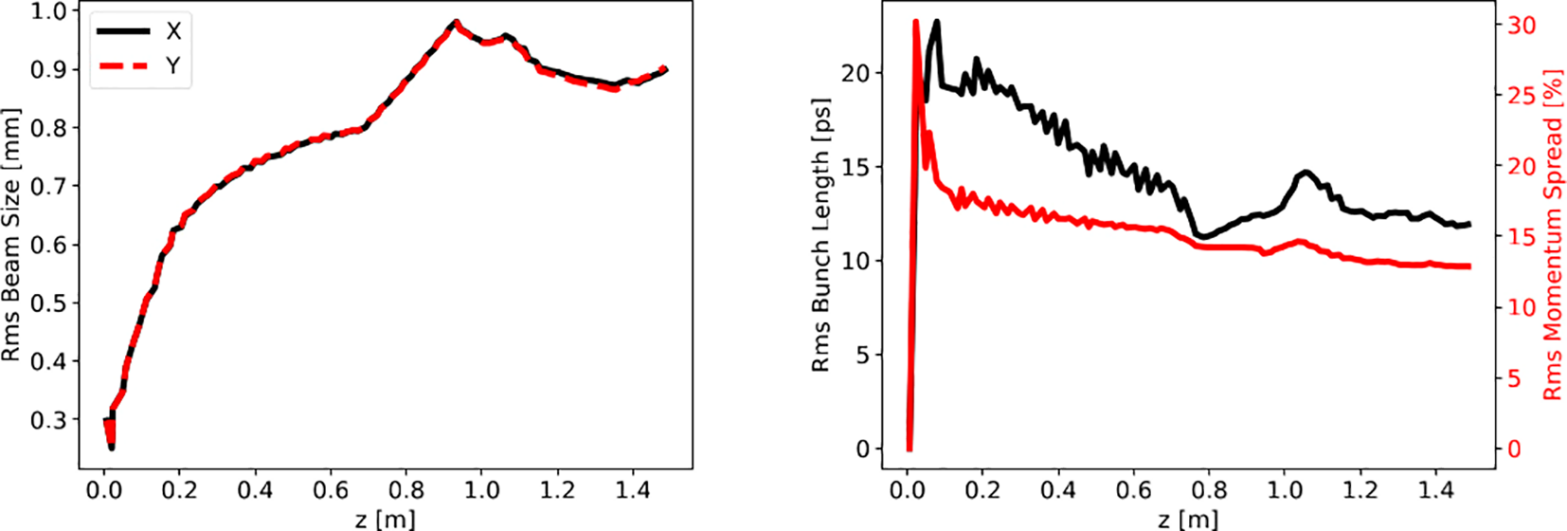
Left: beam envelopes of the accelerating beam. The transverse rms beam size is increased by emittance pressure and space-charge effects. No focusing elements are present on the line except for the RF structures. Right: rms bunch length and momentum spread of the accelerating beam. The final value for the bunch length falls around 13 ps, while the momentum spread at the end of the accelerator is around 15%.

The beam size evolution along the accelerator is determined by two opposite effects: emittance pressure and space-charge, which induce an increase of the beam size in the transverse plane *x* − *y* during the beam propagation, and RF focusing acting in the opposite direction. **Figure 12** (left) shows the rms transverse beam size evolution along the linac.

The beam is initially overfocused as it exits from the cathode and is captured by the SW cavity. Then the beam size increases for the combined effects of space charge (especially at low energy in the SW structure) and emittance pressure, reaching, toward the end of the machine, an rms value of almost saturation equal to 0.8 mm. In the following drift, the beam size increases linearly due to its natural divergence. Finally, in the TW structure, the RF field refocuses the beam while accelerating it.

The longitudinal dynamics of the beam, i.e. the evolution of the bunch length and relative momentum spread is reported in **Figure 12** (right). It is worth specifying that the relative momentum spread, understood as spread of longitudinal momentum, and the relative energy spread, are essentially equivalent concepts for relativistic particles, for the energy is proportional to the momentum via the speed of light in vacuum *c*. The ASTRA simulations for a single bunch are performed assuming a cathode which thermally emits electrons for 175 ps, corresponding to one period of the C-band RF wave. Therefore the bunch length is “zero” at *z* = 0, since no electron has been emitted yet. While electrons are emitted in front of the cathode, they are injected into the SW cavity and a bunch is formed, with its own length and spread of energies. The rms bunch length is slightly reduced during bunching/capture of the beam in the first cells of the SW structure, then it reaches a constant value as the beam accelerates on-crest at relativistic energies in both SW and TW structures.

The rms relative energy spread is approximately constant below 15% in the SW accelerating structure and it is slightly reduced in the TW structure.

#### 4.3.4 Compensation of the energy spread induced by RF pulse shape and beam loading

The powers in the SW and TW accelerating structures needed to obtain the final nominal design beam energy, as discussed above, can be reached after the RF pulse compression in the dedicated SLED cavity.

Before being compressed and distributed to the SW and TW lines, the RF input pulse to the compressor, as it comes out from the klystron, is a square 5 MW pulse, 5 *µ*s long.

In this section, we present the study of the energy spread along the beam due to the combination of the pulse compressor and the beam loading, corresponding to a compression factor of 3 and with *T_RF_* = 1.67 *µ*s. This case is consistent with the pulse compressor design described in Sec. 4.2.2. Such a value for the compression factor has been chosen as a compromise between the compressed length of the RF pulse (to be larger than the current pulse length of 1 *µ*s) and the optimal values of attainable energy gain and spread along the electron current pulse.

**Figure 13:**
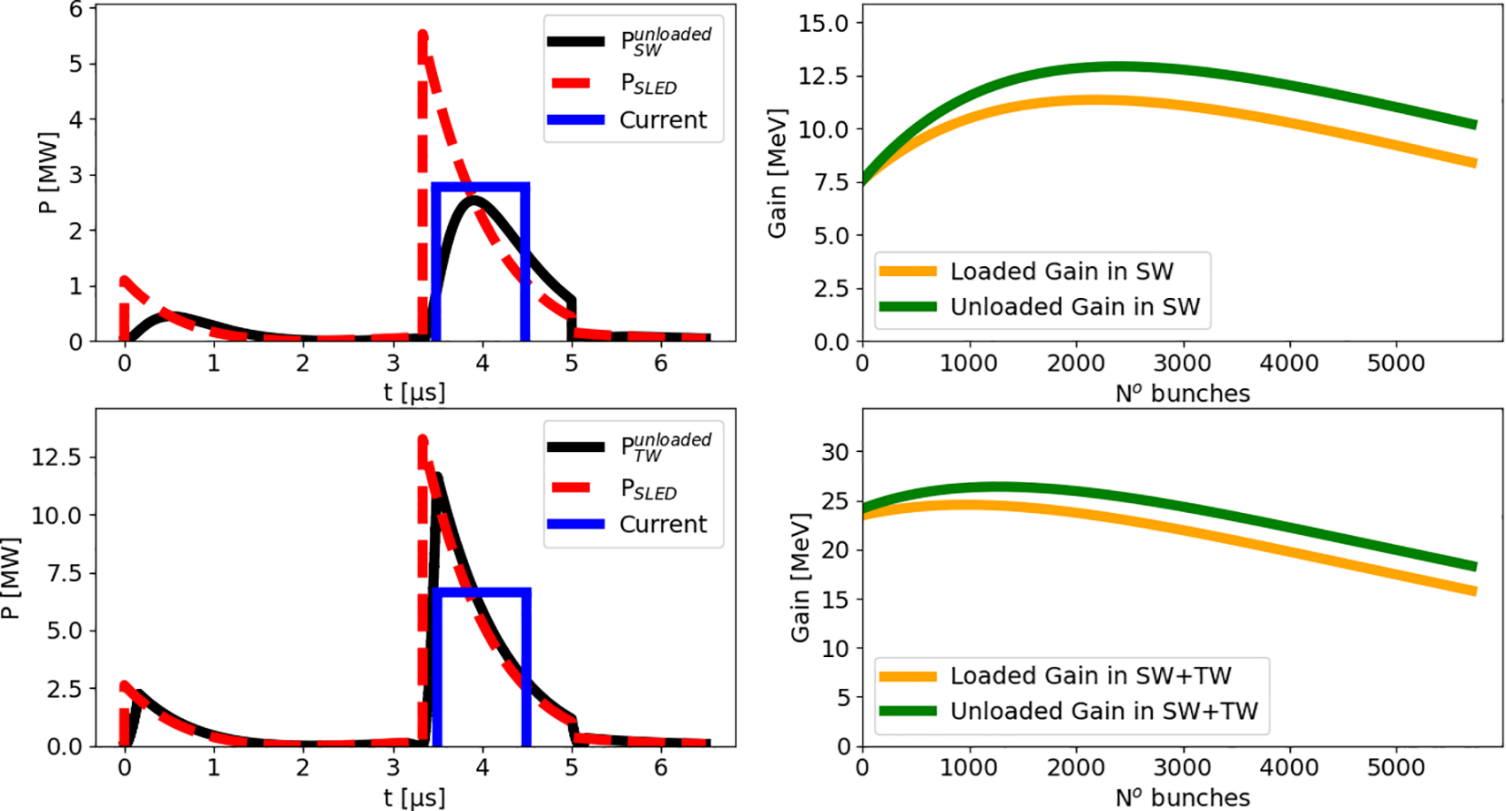
Top-left: compressed pulse (dashed red, *T_RF_* = 1.67 *µ*s) entering the SW cavity, RF pulse shape in the SW cavity (black), temporal position of the 1 *µ*s train of bunches (blue pulse, arbitrary units). Top-right: Bunch energy along the 1 *µ*s train after the SW cavity. Bottom-left: analog to top-left row but for the TW structure. Bottom-right: Bunch energy along the 1 *µ*s train at the end of the accelerator.


Typical shapes for compressed pulses at the exit of the SLED cavity are reported in the left side of **Figure 13** (dashed red lines). They are obtained through analytical calculations based on the equations reported in the following. The pulse entering into any of the accelerating structures, say the SW or the TW, is slightly modified by the time constant of the cavities. However, the typical descending slope of the compressed pulses exiting the SLED is also present in the RF pulses feeding the accelerating structures. To obtain the unloaded power $P_{SW}^{unloaded}$ in the SW cavity (black line in top-left plot of **Figure 13**), the latter can be modeled by a lumped resonant circuit model. We consider a circuit with an inductance $L=\;1/\left(4\pi^{2}\nu_{RF}^{2}C\right)$ , a capacitance $C=Q_{SW}/\left(2\pi \nu_{RF}R_{SW}L_{SW}\right)$ , and a resistance $R_{SW}L_{SW}$ , driven by a current generator that supplies a current *I*(*t*) at frequency $\nu_{RF}$ . The compressed pulse after the SLED cavity, $P_{SLED}$ can be modeled via equations provided by the work of Farkas et al. (40). The circuit equation to be solved for the SW cavity is (21):

(5) $$\frac{\dot{I}}{C}=\ddot{V}+\frac{\dot{V}}{R_{SW}\;L_{SW}C}+4\pi^{2}\nu_{RF}^{2}V$$

where the definition of the generator current is chosen to fit Equation 3, namely:


(6) $$I\left(t\right)=\frac{2\sqrt{\beta_{SW}P_{SLED}\left(t\right)}}{\left(1+\beta_{SW}\right)\sqrt{R_{SW}L_{SW}}}$$

It is worth noting that $P_{SLED}$ here denotes only the fraction of compressed power sent to the SW cavity.

Using the method of the Laplace transform, the low-frequency (neglecting oscillations at *ν_RF_*) analytical solution of Equation 5, with I(t) given by Equation 6, can be easily found. The obtained voltage allows calculating the unloaded SW power dissipated in the cavity defined as:


(7) $$P_{SW}^{unloaded}\left(t\right)=\frac{V^{2}\left(t\right)}{R_{SW}L_{SW}}$$

The power given by Equation 7 is shown in the top-left plot of **Figure 13**, with a black curve.

The maximum energy gain of the beam due to this power in the SW structure would be 14 MeV. However, this value is reduced by the beam loading term that can be expressed as a function of time as (47):


(8) $$\begin{array}{c} \text{Δ}W_{SW}^{bl}\left(t\right)=\frac{\pi eI_{b}R_{SW}L_{SW}}{Q_{SW}}(\frac{1-e^{-\frac{\pi }{Q_{SW}}\left(1+\beta_{SW}\right)\left(\nu_{RF}t+1\right)}}{1-e^{-\frac{\pi }{Q_{SW}}\left(1+\beta_{SW}\right)}}-\frac{1}{2})\;[\theta (t\\ -t_{0,SW})-\theta (t-t_{0,SW}-T_{b})] \end{array}$$

Such a beam energy drop is responsible for a reduction of the maximum energy gain in the SW structure to about 11 *MeV* for the last bunch in the beam current pulse. In Equation 8 we have introduced the Heaviside function *θ*, the injection time of the beam current pulse in the SW structure $t_{0,SW}$ and the duration of the beam current pulse . It is interesting to notice that for $\nu_{RF}t\text{ >}>1,\text{ Δ}W_{SW}^{bl}$ tends to the value expressed by the second term in Equation 3, demonstrating the self-consistency of our analytic approach.

A circuital approach may be significantly more complicated for the study of the RF pulse shape in the TW structure. Therefore, to study the in-cavity pulse shape, we use the expression that describes the unloaded power experienced by a bunch of electrons while traversing the whole TW structure (48):


(9) $$P_{TW}^{unloaded}\left(t\right)=P_{0,TW}{\left[\int_{0}^{L_{TW}}\frac{dz}{L_{TW}}\frac{E_{SLED}\left(t-\frac{z}{v_{g}}\right)}{E_{0}}e^{-\frac{2\pi \nu_{RF}z}{2Q_{TW}v_{g}}}\theta \left(t-\frac{z}{v_{g}}\right)\right]}^{2}$$

where $E_{SLED}$ is the electric field exiting the SLED cavity [calculated by the same model in (40)] and $E_{0}$ its maximum. Moreover, $P_{0,TW}$ is the power available for the TW structure after compression and split.

Equation 9 can be derived from the study of the energy flow in a TW structure. The reduction in beam energy gain due to beam loading in the TW structure as function of time is (48):


(10) $$\text{Δ}W_{TW}^{bl}\left(t\right)=eI_{b}R_{TW}\int_{0}^{L_{TW}}\left[\left(1-e^{-\frac{\pi \nu_{RF}t}{Q_{TW}}}\right)\theta \left(t\right)+\left(e^{-\frac{\pi \nu_{RF}t}{Q_{TW}}}-e^{-\frac{\pi \nu_{RF}z}{Q_{TW}v_{g}}}\right)\theta \left(t-\frac{z}{v_{g}}\right)\right]dz$$

It is possible to verify that in the limit $\nu_{RF}t\text{ >}>1$ , and for low attenuation along the structure $\left(\pi \nu_{RF}L_{TW}/Q_{TW}v_{g}\text{ <}<1\right)$ , Equation 10 tends exactly to the second term in Equation 4. In **Figure 13** the time $t_{0,TW}$ represents the injection time of the beam current pulse in the TW accelerating structure. For the TW structure, the energy drop induced by the beam loading amounts to about 1 MeV, so most of the energy spread distributed along the bunch train is due to the shape of the RF pulse. Synchronizing the start of a $T_{b}=\;1$ *µ*s long electron current pulse with the RF pulse peak in the TW structure would mean accelerating the first bunches of the train to higher energies and the tail bunches to lower energies (due to the RF pulse slope), i.e. inducing energy spread along the bunch train. Indeed, in 1 *µ*s of the RF pulse oscillating at the RF frequency $\nu_{RF}$ = 5.712 GHz, a train of 5712 electron bunches is obtained. Furthermore, another source of energy spread is given by beam loading in the accelerating structures. An optimal injection time of the beam current pulse with respect to the in-cavity RF pulse would flatten to some extent the energy spread induced by the combined action of beam loading and RF pulse shape. The right-hand side of **Figure 13** shows the bunch energy along the 1 *µ*s train corresponding to the optimized injection times $t_{0,SW}$ and $t_{0,TW}$ , for the case of a compression factor equal to 3. For the calculation of the energy gain along the train of bunches in the SW cavity we have used (47):


$$\begin{array}{c} Gain\;in\;SW\;cavity\\ =\frac{2e\sqrt{R_{SW}L_{SW}\beta_{SW}P_{SW}^{unloaded}\left(t=\frac{N}{\nu_{RF}}\right)}}{1+\beta_{SW}}-\text{Δ}W_{SW}^{bl}\left(N\right) \end{array}$$

while for the TW structure, analogously (48):


$$\begin{array}{c} Gain\;in\;TW\;cavity\\ =\sqrt{\frac{2\pi \nu_{RF}L_{TW}}{v_{g}Q_{TW}}R_{TW}L_{TW}P_{TW}^{unloaded}\left(t=\frac{N}{\nu_{RF}}\right)}-\text{Δ}W_{TW}^{bl}\left(N\right) \end{array}$$

We conclude by specifying that the power fractions 30% and 70% in **Figure 13** refer to the total available power reduced by 25%, in order to consider both possible losses along the transmission line and power attenuation, the latter exploited to maintain the average final electron energy to a maximum of 24 MeV.

## 5 Dose profiles

**Figure 14:**
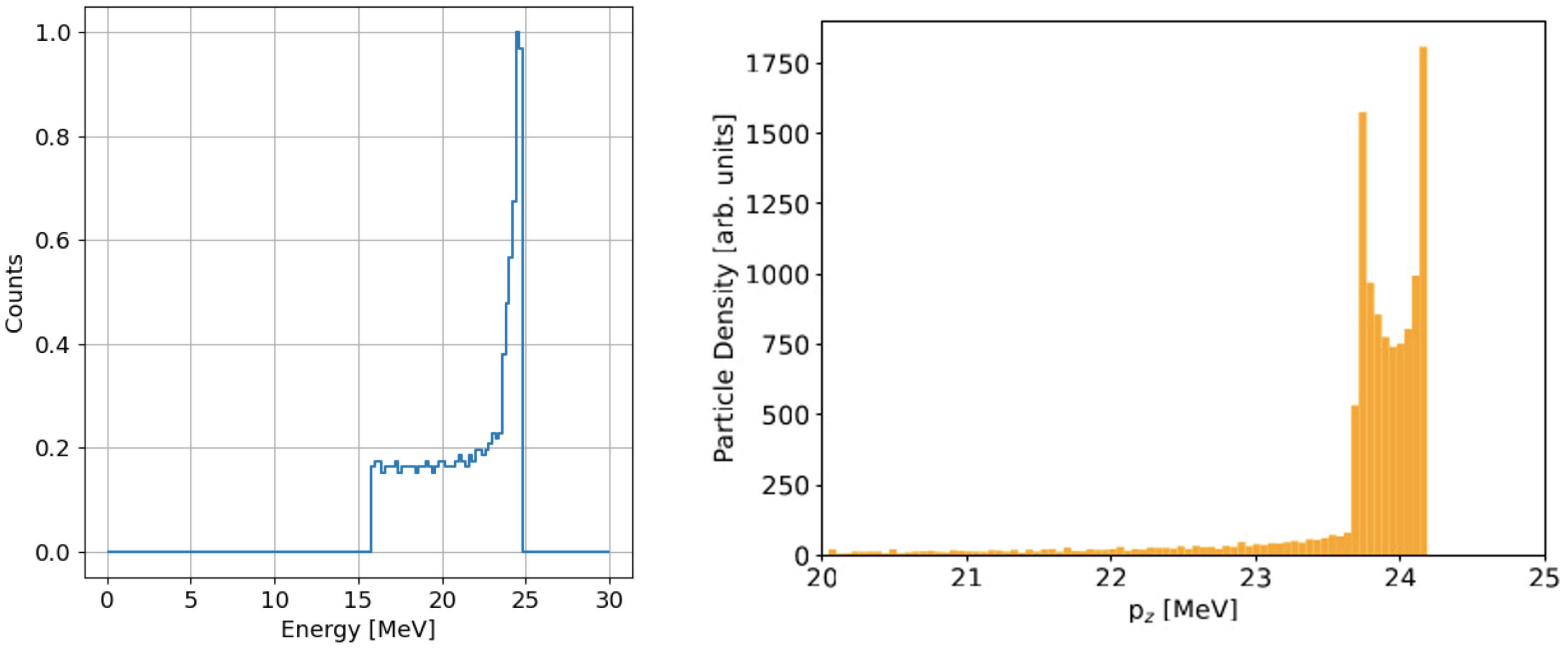
Left: energy distribution of a train of bunches with an RF pulse duration of 1.67 *µ*s. The number of counts is normalized to its maximum. Right: energy spectrum of the single bunch simulated by ASTRA at the exit of the accelerator.

As a reference for the dose distributions delivered to a patient, we considered the corresponding ones in water produced by a train of bunches with the energy distribution shown in **Figure 14** (left), obtained as a histogram of the bottom-right plot in **Figure 13** with the SLED compression factor discussed in the previous section. This spectrum represents the distribution of the mean energy of the single bunches contained in the current pulse. For simplicity, the single bunch energy spread (Energy $≃p_{z}c$ ) of the kind shown in the right-end side of **Figure 14** has been neglected since it constitutes only a small correction to the final electron energy distribution.

**Figure 15:**
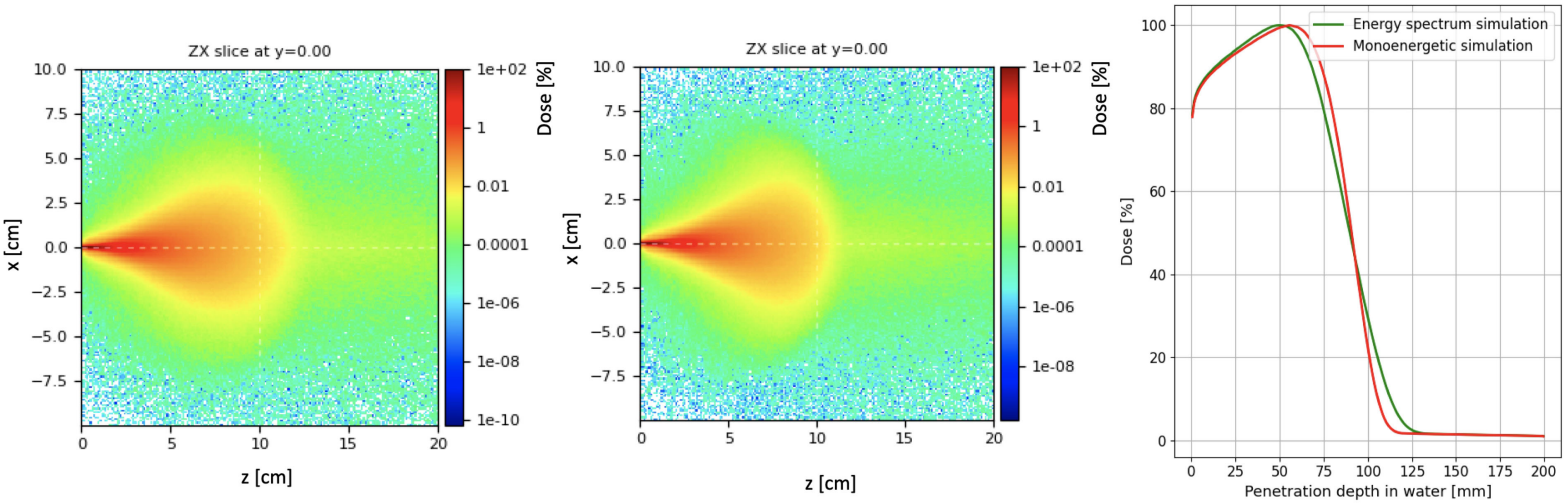
Normalized map dose in logarithmic scale considering the energy spectrum reported in **Figure 14** (left) and with monoenergetic beam (center). Percentage dose profile as a function of the penetration depth in water (right) for the two energy spectra.

This energy spectrum directly influences the dose distribution delivered to the patient. As a case study, we analyzed the dose deposition in a water volume, representing patient tissue, using simulations performed with FLUKA. The resulting 2D dose distribution is shown on the left-end side of **Figure 15**, presented on a logarithmic scale. For comparison, the center of the same figure illustrates the case of a monoenergetic beam with energy equal to the mean energy of the spectrum shown in **Figure 14** (left), equal to 21.13 MeV. The small initial transverse size, around 1-2 mm FWHM, is due to the fact that the linac produces a pencil beam at the exit of the TW. However, electron diffusion in water, caused by multiple scattering and photon production by means Bremsstrahlung process, dominates the beam’s transverse spread after just a few cm of depth.

The longitudinal integrated dose distributions for the same cases are presented on the right-hand side of the same figure. The green line represents the dose profile from the simulation using the energy spectrum of **Figure 14** (left), while the red line corresponds to the dose obtained with the mono-energetic beam. The dose peak, which differs by a few percent compared to the mono-energetic case (3.69·10^8^ and 3.71·10^8^ Gy/primary for the energy distribution and mono-energetic beam simulations, respectively), appears in the real case to be shifted a few millimeters backward relative to the mono-energetic beam, due to the contribution of low-energy electrons.

## 6 Conclusions and future developments

This paper presents the design of a VHEE linac prototype to be built at the University of Rome La Sapienza, with two primary objectives: testing the acceleration scheme and associated technologies (C-band SW structure followed by a C-band TW structure) and providing UHDR electron beams to explore FLASH irradiation techniques and conduct radiobiological experiments. It also represents a foundational step toward the development of a compact VHEE linac capable of reaching 100 MeV energy levels.

Future integration of VHEE linacs into clinical settings will require targeted research and development to address key challenges. These include optimizing treatment planning systems for FLASH delivery, establishing reliable quality assurance protocols, and improving patient positioning and immobilization techniques. Additionally, extensive clinical trials will be crucial to understanding the long-term effects of FLASH radiotherapy with VHEE beams on human health.

## Data Availability

The raw data supporting the conclusions of this article will be made available by the authors, without undue reservation.

## References

[1] Limoli CL Vozenin MC . Reinventing radiobiology in the light of flash radiotherapy. Annu Rev Cancer Biol. (2023) 7:1–21. doi: 10.1016/j.radonc.2019.04.008 39421564 PMC11486513

[2] Montay-Gruel P Petersson K Jaccard M Boivin G Germond JF Petit B . Irradiation in a flash: Unique sparing of memory in mice after whole brain irradiation with dose rates above 100gy/s. Radiotherapy Oncol. (2017) 124:365–9. doi: 10.1016/j.radonc.2017.05.003 28545957

[3] Montay-Gruel P . Hypofractionated flash-rt as an effective treatment against glioblastoma that reduces neurocognitive side effects in mice. Clin Cancer Res. (2021) 27:75–784. doi: 10.1158/1078-0432.CCR-20-0894 PMC7854480 33060122

[4] Montay-Gruel P . Long-term neurocognitive benefits of flash radiotherapy driven by reduced reactive oxygen species. Proc Natl Acad Sci U. S. A. (2019) 166:10943–51. doi: 10.1073/pnas.1901777116 PMC6561167 31097580

[5] Alaghband Y . Neuroprotection of radiosensitive juvenile mice by ultra-high dose rate flash irradiation. Cancers. (2020) 12:1–21. doi: 10.3390/cancers12061671 PMC7352849 32599789

[6] Limoli CL . The sparing effect of flash-rt on synaptic plasticity is maintained in mice with standard fractionation. Radiother. Oncol. (2023) 186:109767. doi: 10.1016/j.radonc.2023.109767 37385377 PMC11045040

[7] Mascia AE . Proton flash radiotherapy for the treatment of symptomatic bone metastases: The fast-01 nonrandomized trial. JAMA Oncol. (2023) 9:62–9. doi: 10.1001/jamaoncol.2022.5843 PMC9589460 36273324

[8] Rohrer Bley C . Dose- and volume-limiting late toxicity of flash radiotherapy in cats with squamous cell carcinoma of the nasal planum and in mini pigs. Clin Cancer Res. (2022) 28:3814–23. doi: 10.1158/1078-0432.CCR-22-0262 PMC9433962 35421221

[9] Vozenin MC . The advantage of flash radiotherapy confirmed in mini-pig and cat-cancer patients. Clin Cancer Res. (2019) 25:35–42. doi: 10.1158/1078-0432.CCR-17-3375 29875213

[10] Faillace L Alesini D Bisogni G Bosco F Carillo M Cirrone P . Perspectives in linear accelerator for flash vhee: Study of a compact c-band system. Physica Med. (2022) 104:149–59. doi: 10.1016/j.ejmp.2022.10.018 36427487

[11] Palumbo L Sarti A Mostacci A De Gregorio A De Arcangelis D Francescone D . Safest. a compact c-band linear accelerator for vhee-flash radiotherapy. J OF Phys. (2023) 2687:5079–82. doi: 10.18429/JACoW-IPAC2023-THPM087

[12] Bazalova-Carter M Qu B Palma B Hårdemark B Hynning E Jensen C . Treatment planning for radiotherapy with very high-energy electron beams and comparison of vhee and vmat plans. Med Phys. (2015) 42:2615–25. doi: 10.1118/1.4918923 25979053

[13] Schüler E Eriksson K Hynning E Hancock SL Hiniker SM Bazalova-Carter M . Very high-energy electron (vhee) beams in radiation therapy; treatment plan comparison between vhee, vmat, and ppbs. Med Phys. (2017) 44:2544–55. doi: 10.1002/mp.12233 28339108

[14] Sarti A De Maria P Battistoni G De Simoni M Di Felice C Dong Y . Deep seated tumour treatments with electrons of high energy delivered at flash rates: The example of prostate cancer. Front Oncol. (2021) 11:777852. doi: 10.3389/fonc.2021.777852 35024354 PMC8744000

[15] Muscato A Arsini L Battistoni G Campana L Carlotti D De Felice F Battistoni G De Gregorio A De Maria P Fischetti M Franciosini G . Treatment planning of intracranial lesions with vhee: comparing conventional and flash irradiation potential with state-of-the-art photon and proton radiotherapy. Front Phys. (2023) 11:1185598. doi: 10.3389/fphy.2023.1185598

[16] De Simoni M Battistoni G De Gregorio A De Maria P Fischetti M Franciosini G . A data-driven fragmentation model for carbon therapy gpu-accelerated monte-carlo dose recalculation. Front Oncol. (2022) 12:780784. doi: 10.3389/fonc.2022.780784 35402249 PMC8990885

[17] Schiavi A Senzacqua M Pioli S Mairani A Magro G Molinelli S . Fred: a GPU-accelerated fast-monte carlo code for rapid treatment plan recalculation in ion beam therapy. Phys Med Biol. (2017) 62:7482–504. doi: 10.1088/1361-6560/aa8134 28873069

[18] Franciosini G Battistoni G Cerqua A De Gregorio A De Maria P De Simoni M . Gpu-accelerated monte carlo simulation of electron and photon interactions for radiotherapy applications. Phys Med Biol. (2023) 68:044001. doi: 10.1088/1361-6560/aca1f2 36356308

[19] Kokurewicz K Brunetti E Curcio A Gamba D Garolfi L Gilardi A . An experimental study of focused very high energy electron beams for radiotherapy. Commun Phys. (2021) 4:33. doi: 10.1038/s42005-021-00536-0

[20] El-Ashmawy M . Overall quality comparison of c-band and x-band medical linacs, In: Proceedings of the 14th Symposium on Accelerator Science and Technology, November 11-13, 2003, Tsukuba, Japan. (2003). pp. 1–3.

[21] Wangler T . Rf linear accelerators. Wiley New York. (1998). doi: 10.1002/9783527623426

[22] Hanna MS . Applications of X-band technology in medical accelerators, In: Proceedings of the 1999 Particle Accelerator Conference, New York City, March 29th - April 2nd, 1999. (1999). pp. 2516–8.

[23] Kilpatrick WD . Criterion for vacuum sparking designed to include both rf and dc. Rev Sci Instrum. (1957) 28:824. doi: 10.1063/1.1715731

[24] Tanabe E Wang JW Loew GA . Voltage breakdown at x-band and c-band frequencies. Proc 1986 Int Linac Conf. (1986) 860602:458–60. Available online at: https://inspirehep.net/literature/240748.

[25] Wang J Loew G . Field emission and rf breakdown in high-gradient room temperature linac structures. Tech. rep. Stanford Univ. Stanford Linear Accelerator Center CA (US). (1997). doi: 10.2172/663321

[26] Higo T Higashi Y Matsumoto S Yokoyama K Doebert S Grudiev A . Advances in x-band tw accelerator structure operating in the 100 mv/m regime. Proc IPAC’10 Kyoto Japan. (2010) 2010:3702–4. Available online at: https://accelconf.web.cern.ch/IPAC10/papers/thpea013.pdf.

[27] Simakov EI Dolgashev VA Tantawi SG . Advances in high gradient normal conducting accelerator structures. Nucl Instruments Methods Phys Res Section A: Accelerators Spectrometers Detectors Associated Equip. (2018) 907:221–30. doi: 10.1016/j.nima.2018.02.085

[28] Dolgashev VA Faillace L Spataro B Tantawi S Bonifazi R . High-gradient rf tests of welded *x*-band accelerating cavities. Phys Rev Accel. Beams. (2021) 24:081002. doi: 10.1103/PhysRevAccelBeams

[29] Sakurai T Ego H Inagaki T Asaka T Suzuki D Miura S . *c*-band disk-loaded-type accelerating structure for a high acceleration gradient and high-repetition-rate operation. Phys Rev Accel. Beams. (2017) 20:42003. doi: 10.1103/PhysRevAccelBeams.20.042003

[30] Fang W Gu Q Zhao Z Tong D . The c-band traveling-wave accelerating structure for compact x-fel at sinap, In: Proceedings of the second International Particle Accelerator Conference, 4 to 9 September, 2011, San Sebastián, Spain (2011). pp. 133–5.

[31] Alesini D Bellaveglia M Biagini M Boni R Brönnimann M Cardelli F . Design, realization and test of c-band accelerating structures for the sparc lab linac energy upgrade. Nucl Instruments Methods Phys Res A. (2016) 837:161–70. doi: 10.1016/j.nima.2016

[32] Alesini D Bellaveglia M Gallo BSA Lollo V Pellegrino L Piersanti L . Design of high gradient, high repetition rate damped c-band rf structures. Phys Rev Accelerators Beams. (2017) 20:032004-1–032004-20. doi: 10.1103/PhysRevAccelBeams.20.032004

[33] Alesini D Boni R Pirro GD Raddo RD Ferrario M Gallo A . The c-band accelerating structures for sparc photoinjector energy upgrade. J Instrumentation. (2013) 8:P05004. doi: 10.1088/1748-0221/8/05/P05004

[34] Tantawi S Nasr M Li Z Limborg C Borchard P . Design and demonstration of a distributedcoupling linear accelerator structure. Phys Rev Accel. Beams. (2020) 23:92001. doi: 10.1103/PhysRevAccelBeams.23.092001

[35] Nasr M Nanni E Breidenbach M Weathersby S Oriunno M Tantawi S . Experimental demonstration of particle acceleration with normal conducting accelerating structure at cryogenic temperature. Phys Rev Accel. Beams. (2021) 24:93201. doi: 10.1103/PhysRevAccelBeams.24.093201

[36] Miller RH . Comparison of standing-wave and traveling-wave structures. SLAC-PUB. (1986) 3935:200–5. Available online at: https://digital.library.unt.edu/ark:/67531/metadc1063921/.

[37] Giuliano L Carillo M Chiadroni E De Gregorio A Ficcadenti L Francescone D . Safest project, a compact c-band rf linac for vhee flash radiotherapy, In: Proceedings of the International Particle Accelerator Conference, 19-24 May 2024, Nashville TN. JACoW Publishing, Proc. IPAC 24. (2024) pp. 3643–6. doi: 10.18429/JACoW-IPAC2024-THPR55

[38] Xiang S Yue Y Yuzheng L . Rf phase focusing and asymmetric field shape in standing-wave electron linacs, In: Proceedings of the first Asian Particle Acceleration Conference APAC 1998, Tsukuba, Japan, March 23 to 27, 1998. (1998). pp. 184–6.

[39] Rosenzweig J Serafini L . Transverse particle motion in radio-frequency linear accelerators. Phys Rev E. (1994) 49:1599–602. doi: 10.1103/PhysRevE.49.1599 9961373

[40] Farkas Z Hogg H Loew G Wilson PB . Sled: A method of doubling slac’s energy, In: Proceedings of 9th International Conference on the High-Energy Accelerators (HEACC 1974), Stanford, California, May 2-7, 1974. (1974). p. 576.

[41] Multiphysics C . Introduction to comsol multiphysics^®^ . In: COMSOL Multiphysics. Burlington, MA: COMSOL. (1998). p. 32.

[42] CST (2022). Available online at: https://www.3ds.com/products-services/simulia/products/cst-studio-suite/ (Accessed February 25, 2025).

[43] Faillace L Barone S Battistoni G Di Francesco M Felici G Ficcadenti L . Compact *s*-band linear accelerator system for ultrafast, ultrahigh dose-rate radiotherapy. Phys Rev Accel. Beams. (2021) 24:50102. doi: 10.1103/PhysRevAccelBeams.24.050102

[44] Giuliano L Bosco F Carillo M Felici G Ficcadenti L Mostacci A . Rf design and measurements of a c-band prototype structure for an ultra-high dose rate medical linac. Microwave Measurements Methods Instruments Science Soc Industry. (2023) 7:10. doi: 10.3390/instruments7010010

[45] Alesini D Alessandro G Bruno S Marinelli A Palumbo L . Design of couplers for traveling wave rf structures using 3d electromagnetic codes in the frequency domain. Nucl Instruments Methods Phys Res Section A Accelerators Spectrometers Detectors Associated Equip. (2007) 580:1176–83. doi: 10.1016/j.nima.2007.06.045

[46] Flottmann K . Astra: A space charge tracking algorithm(2022). Available online at: http://www.desy.de/~mpyflo (Accessed February 25, 2025).

[47] Boussard D . Beam loading. CERN-SPS-86-10-ARF. (1987). Available online at: https://cds.cern.ch/record/167557.

[48] Lunin A Yakovlev V Grudiev A . Analytical solutions for transient and steady state beam loading in¡? format?¿ arbitrary traveling wave accelerating structures. Phys Rev Special Topics—Accelerators Beams. (2011) 14:052001. doi: 10.1103/PhysRevSTAB.14.052001

